# Potency of spirulina (*Spirulina platensis*) on arsenic-induced lipid peroxidation in rat

**DOI:** 10.5455/javar.2021.h519

**Published:** 2021-06-27

**Authors:** Abul Khair, Md. Abdul Awal, Md. Shafiqul Islam, Md. Zahorul Islam, Damanna R. Rao

**Affiliations:** 1 Department of Pharmacology, Faculty of Veterinary Science, Bangladesh Agricultural University, Mymensingh-2202, Bangladesh; 2 Department of Livestock Services, Quality Control Laboratory, Savar, Dhaka, Bangladesh; 3 National Institute of Food and Agriculture-USDA, Washington DC, USA

**Keywords:** Inorganic, arsenic, lipid peroxidation, rats, spirulina, total protein

## Abstract

**Objective:**

Natural substances found in dietary sources and medicinal plants have attracted considerable attention in recent years as chemopreventive agents. Spirulina is a blue-green alga that possesses chemopreventive properties. The purpose of this study was to determine the effect of spirulina on rats with inorganic arsenic (As) [sodium arsenite (NaAsO_2_)]-induced lipid peroxidation.

**Materials and Methods:**

120 rats were randomly assigned to 10 groups and designated T0, T1, T2, T3, T4, T5, T6, T7, T8, and T9. One group was kept as a control (T0) that received no treatment. The seven groups received 3.0 mg of NaAsO_2_/kg body weight in drinking water and were given spirulina *ad libitum*. T1 was treated with NaAsO_2_ but not with spirulina. Two groups of rats (T2 and T3), on the other hand, were treated with spirulina without receiving any As (NaAsO_2_). T2 received agro-based spirulina (Ab-Sp; grown in 1.5% soybean meal media and harvested on day 12 of seed inoculation) at 2.0 gm/kg feed, whereas T3 received commercially available spirulina (Com-Sp) at 2.0 gm/kg feed. T4, T5, and T6 were concurrently treated with Ab-Sp at 1.0, 1.5, and 2.0 gm/kg of feed. On the other hand, T7, T8, and T9 induced by NaAsO_2_ were concurrently treated with Com-Sp at 1.0, 1.5, and 2.0 gm/kg feed. All groups received treatment for 90 days.

**Results:**

The efficacy of both spirulina in preventing lipid peroxidation caused by As was determined quantitatively by measuring the rats’ serum malondialdehyde (MDA). The results indicated that As supplementation increased serum MDA levels, whereas both types of spirulina significantly decreased them. The highest dose of Ab-Sp (2.0 gm/kg feed) was found to be the most effective in preventing lipid peroxidation in rats treated with inorganic As.

**Conclusion:**

Ab-Sp could be a natural, cost-effective, and safe measure to mitigate As toxicity.

## Introduction

Arsenic (As) is an omnipresent environmental contaminant that can affect the physiological function of both humans and animals [1]. Inorganic arsenicals have carcinogenic and mutagenic effects and are harmful to endogenous physiological processes [2]. On the contrary, organic As compounds have insignificant effects on human and animal bodies [3]. Chronic As poisoning resulting from contaminated drinking water and food is causing numerous problems on human and animal health in Bangladesh and its surrounding countries [1]. In developing countries like Bangladesh and India, many peoples are consuming As through contaminated food chains and water [4,5]. Many people are exposed to As levels greater than 10.0 μg/l due to contaminated drinking water [5]. Although drinking water is the most common source of chronic As poisoning in people [6], As-contaminated food could potentially be a significant source [7].

Lipid peroxidation is the oxidative degradation of polyunsaturated fatty acids and is a general mechanism of cell death and cytotoxicity. As a result, reactive oxygen species (ROS) levels rise [8]. Chronic low-level As exposure may increase the production of ROS, which can cause the oxidation of cellular lipids, deoxyribonucleic acid (DNA), and proteins [9]. Malondialdehyde (MDA) is the primary indicator of lipid peroxidation and is mutagenic and carcinogenic [10]. As causes lipid peroxidation and apoptosis in a broad range of cell types [11]. As-induced oxidative damage causes several health issues, including melanosis, hyperkeratosis, black foot disease, hepatomegaly, neuropathy that progresses to cancer or gangrene, liver fibrosis, and cardiovascular diseases, like ischemic heart disease, acute myocardial infarction, hypertension, and atherosclerosis [12].

To protect the integrity of cells or tissues, a suite of antioxidant chemicals and enzyme systems neutralize ROS release. Toxic substances that generate oxidative stress can disrupt the delicate equilibrium between antioxidants and ROS formation [14]. Oxidative damage to proteins, lipids, and DNA occurs when the ROS concentrations are not neutralized by internal antioxidants and oxygen radical scavenging enzymes, resulting in cytotoxicity, genotoxicity, and even carcinogenesis [14].

Spirulina (*Spirulina platensis*) is a blue-green alga, considered a complete food [15]. Spirulina possesses anti-nephrotoxic effects caused by heavy metals and pharmaceuticals [16]. It is high in protein levels, including vital amino acids, antioxidants, and phytochemicals that activate enzyme systems to help in As detoxification [17]. Spirulina contains considerable amounts of protein, fat, carbohydrate, vitamins, minerals, chlorophyll, carotenoid, phycocyanin, and other pigments that are beneficial to health. Spirulina’s cell wall lacks cellulose, making it a suitable meal for humans and animals [15].

Spirulina has various therapeutic effects, including cholesterol and cancer reduction, immune system enhancement, increased intestinal lactobacilli, and reduced nephrotoxicity, caused by heavy metals, drugs, and radiation [18]. Spirulina or its extracts have been shown to prevent or inhibit oxidative stress and hepatic damage caused by drug abuse and heavy metal exposure [19], as well as inflammation and cell degeneration in both humans and animals [18]. Additionally, spirulina benefits cardiovascular disease, Parkinson’s disease, malnutrition, sclerosis, and wound healing [18]. Spirulina has antiarthritic properties due to the presence of phycocyanin. Also, it has anti-atherogenic and tumor-inhibiting properties, chemo- and radioprotective properties [17,19].

By chelating As and scavenging free radicals, antioxidants can help reduce As toxicity [20]. Thus, supplementation with potentially beneficial antioxidants appears to be helpful in the treatment of arsenicosis. Spirulina has been shown to bind and remove heavy metals [20]. Thus, spirulina consumption can help mitigate As’s toxic effects by reducing oxidative stress, inhibiting lipid peroxidation, and decreasing susceptibility to arsenicosis by addressing malnutrition. This study aimed to determine the efficacy of spirulina in preventing lipid peroxidation during As toxicity in rats.

## Materials and Methods

### Ethical approval of laboratory animals

The laboratory experimental rats were bred and humanely sacrificed following the guidelines set by the Animal Welfare and Experimental Ethics Committee of Bangladesh Agricultural University [approval number: AWEEC/BAU/2021(06)].

### Animals and treatments

One hundred twenty male Long Evans rats weighing between 250 and 300 gm (BW) were used in this study. The rats were randomly assigned to 10 groups of 12 rats in each group: T0, T1, T2, T3, T4, T5, T6, T7, T8, and T9. One group was kept as a negative control (T0) without any treatment, while another was kept as a positive control (T1) with 3.0 mg sodium arsenite (NaAsO_2_)/kg BW in drinking water daily. Individual rats in the T2 and T3 groups received the highest dose (2.0 gm/kg feed) of agro-based spirulina (Ab-Sp) (grown in 1.5% soybean meal media and harvested on day 12 after seed inoculation) or commercially available spirulina (Com-Sp) with feed. The rats in groups T4, T5, T6, T7, T8, and T9 received 3.0 mg NaAsO_2_/kg BW in drinking water. Simultaneously, different concentrations of Ab-Sp (T4, T5, and T6) and Com-Sp (T7, T8, and T9) were fed to As-treated rats. All groups received treatment for a total of 90 days.

### Preparation and feeding of the treatment components

A 0.2% NaAsO_2_ stock solution was prepared by dissolving 2.0 g of NaAsO_2_ in 1 l of deionized water (DW) and stored at 4°C for a maximum of 7 days to feed the trial rats. Individual 100-ml acid-washed and dried glass beakers were filled with the required doses (1.0, 1.5, and 2.0 gm/kg feed) of both spirulina species (Ab-Sp and Com-Sp). Next, spirulina was combined with DW in the beaker to create a thin spirulina suspension. Then, the individual doses of spirulina suspension were combined with the required amount (1.0 kg) of commercial pellet broiler finisher feed. This task was accomplished by dribbling the spirulina suspension onto the feed and stirring simultaneously with a glass rod, resulting in a firm adhesion of the spirulina to the pellets. The spirulina-infused feeds were then placed in a stainless steel tray and dried for at least 20 h at 50°C in an electric oven. Following preparation, the dry spirulina mixed feed was immediately transferred to an airtight food-grade polypropylene container and given to the trial rats for 5 days. About 12 h before day 5 after the previous batch’s production, a new batch of spirulina mixed feed was made and dried.

NaAsO_2_ was given in drinking water every morning (half of a group’s daily water intake). The rats were given unrestricted access to normal drinking water after swallowing the entire volume of NaAsO_2_ solution. Simultaneously, all six spirulina-treated groups received *ad libitum* spirulina mixed meal comprising appropriate spirulina doses (Ab-Sp and Com-Sp) (T2, T3, T4, T5, T6, T7, T8, and T9).

### Sampling

Four samplings were conducted at 30-day intervals, namely on Day 0, Day 30, Day 60, and Day 90. On each sampling day, three rats were randomly selected from each group and placed in an airtight chamber to undergo general inhalation anesthesia with chloroform (Fisher Scientific UK Limited, UK). After complete anesthesia, approximately 4.0–5.0 ml of blood was collected directly from each rat’s heart using a 10.0-ml disposable syringe and transferred to a 10-ml centrifuge tube. Blood was maintained at room temperature for 15 min before being stored at 4°C overnight. The following morning, the blood samples were brought to room temperature and centrifuged for 15 min at 1,500 rpm. The serum supernatant was collected into sterilized Eppendorf tubes and stored at –20°C until testing.

### Determination of lipid peroxidation

#### Determination of total serum protein (TSP)

The TSP concentration was determined quantitatively using the biuret end-point method with a diagnostic kit (DiaSys Diagnostic System GmbH, Holzheim, Germany), as described by Tietz [27]. Proteins formed a violet color complex with the kit reagent’s copper ions in the reagent’s alkaline solution. Because the absorbance of the color produced in the reaction is directly proportional to the sample’s protein concentration, the sample’s absorbance and standard were determined within 60 min using a spectrophotometer set to 546 nm, optical path 1 cm, and 37°C. TSP was calculated from the absorbance of the sample (∆A_Sample_) in respect to an absorbance of the standard (∆A_Standard_), and the result was multiplied by the concentration of the standard as follows:

Total protein (gm/dl) = (∆A_Sample_/∆A_Standard_) × Conc. of standard (gm/dl).

#### Determination of serum MDA

**Figure 1.**
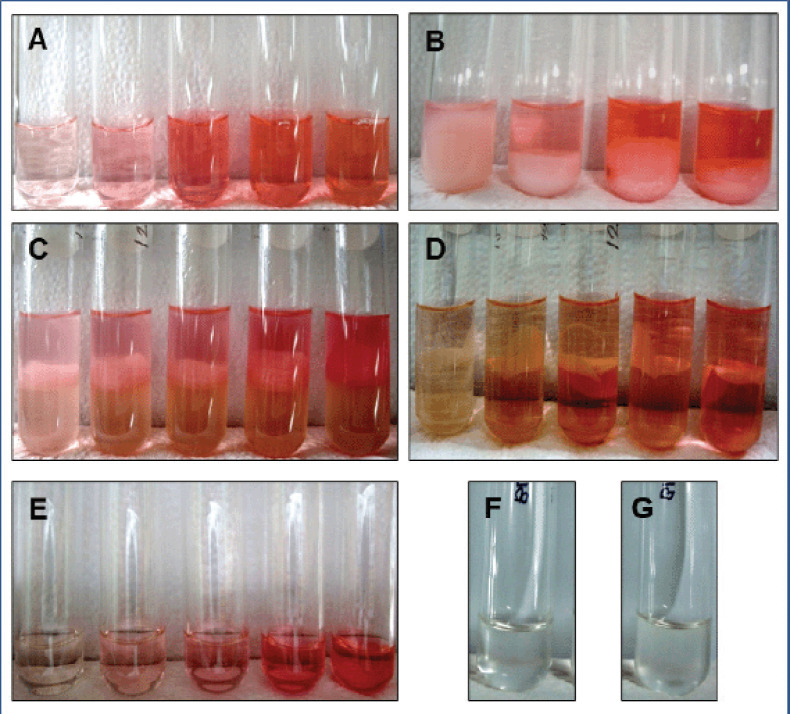
Different steps of serum MDA determination. (A) Samples before heating, (B) samples after heating (turbidity), (C) after adding iso-butanol, (D) after centrifugation, (E) finally collected samples, (F) finally collected reagent blank, and (G) finally collected standard blank.

MDA levels in serum samples were quantified using the method described by Ohkawa et al. [26]. To summarize, 300.0 μl of serum was separated into 10-ml heat-resistant glass test tubes, and 900.0 μl of 20% (w/v) trichloroacetic acid (TCA; C_2_HCl_3_O_2_; Sigma-Aldrich, St. Louis, MO) was added to each sample. The individual sample was then vortexed for 5 min with 1,500.0 μl of 0.8% (w/v) 2-thiobarbituric acid (2-TBA; C_4_H_4_N_2_O_2_S; Sigma-Aldrich, St. Louis, MO) in 1.1% (w/v) sodium dodecyl sulfate (NaC_12_H_25_SO_4_; Sigma-Aldrich, St. Louis, MO) (Fig. 1A). After heating the mixtures in a water bath at 95°C for 60 min, the reaction was halted by cooling the tubes in an ice tray (Fig. 1B). The sample mixture’s red pigments were extracted by adding 2,700.0 μl of iso-butanol (Merck, Darmstadt, Germany) in a 1:1 ratio and vortexed for 5 min. After centrifugation at 4,000 rpm for 10 min (Fig. 1D), the tubes were collected and the organic layer of the supernatant was collected (Fig. 1E). A reagent blank (Fig. 1F) was prepared in the same manner.

Exactly 2.0 mmol of 1, 1, 3, 3 tetraethoxypropane (C_11_H_24_O_4_; Sigma-Aldrich, St. Louis, MO) was dissolved in 1,000.0 ml of DW (2,000,000 nmol/ml) to form a stock standard solution. Just prior to use, the stock solution was diluted 1,000-fold (2,000 nmol/ml) and working standards were prepared at concentrations of 0.0, 2.0, 4.0, 6.0, 8.0, 10.0, and 12.0 nmol/ml. A standard blank (Fig. 1G) was prepared in the same manner as a sample was prepared. The absorbance of the standards was measured against the standard blank, and that of the samples was measured against the reagent blank using a spectrophotometer set to 532 nm. The absorbance versus concentrations of the standards was used to create a calibration curve. The concentration of MDA in nmol/ml of serum was calculated by plotting the absorbance readings of the samples against the standard curve. Because the thiobarbituric acid reactive substance (TBARS) assay values are typically expressed in terms of MDA equivalents and the TBARS content was always expressed in nmol/mg of protein, the MDA concentrations in the samples were finally expressed as nmol/mg of protein by dividing the MDA concentration (nmol/ml) by the amount of TSP.

### Statistical analysis 

The data were analyzed using the Statistical Package for the Social Sciences version 10.0. The values’ mean and standard deviation were computed, and a one-way analysis of variance was used to establish the significance of the differences between them. The statistical significance was determined using a *p*-value of 0.05.

## Results

### Total serum protein (TSP)

**Figure 2.**
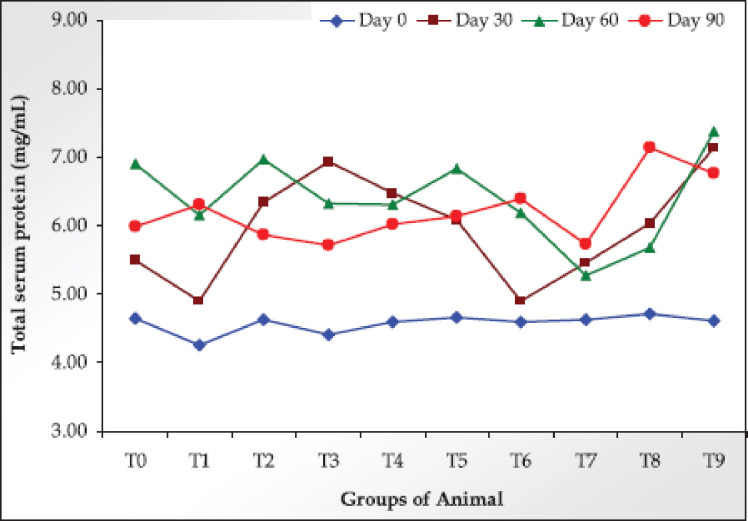
Values of TSP in the trial rats on different sampling days.

TSP values are presented in Table 1. On day 0, TSP values of all groups were from 4.25 ± 0.05 to 4.71 ± 0.38 mg/ml. NaAsO_2_ induction significantly (*p *< 0.01) decreased the values of TSP in rats on day 30 and day 60. Both the Ab-Sp and Com-Sp treatments significantly (*p *< 0.01) increased TSP values in rats on day 30 and day 60. The TSP values of all the doses of the Ab-Sp were significantly (*p *< 0.01) increased on day 30 and day 60 compared to the As control group, but that were increased with 1.0 gm Ab-Sp/kg feed and decreased with the other two doses of the Ab-Sp on day 90. However, the values did not differ significantly compared to the As control group. The effects of Com-Sp were almost similar to that of Ab-Sp in the respective doses and sampling days (Fig. 2; Table 1).

**Table 1.** Values of TSP in the trial rats on different sampling days.

Groups of animal	TSP (mg/ml)			
	Day 0	Day 30	Day 60	Day 90
T0: Control	4.64 ± 0.55	5.49 ± 0.56^bc^	6.90 ± 0.70^ab^	5.98 ± 0.67
T1: As 3 mg/kg BW	4.25 ± 0.05	4.89 ± 0.01^c^	6.16 ± 1.68^ab^	6.31 ± 0.55
T2: Ab-Sp (2 gm/kg feed)	4.62 ± 0.47	6.34 ± 0.63^abc^	6.96 ± 1.13^ab^	5.86 ± 2.27
T3: Com-Sp group (2 gm/kg feed)	4.40 ± 0.37	6.93 ± 0.31^ab^	6.33 ± 0.12^ab^	5.71 ± 0.48
T4: As + Ab-Sp (1 gm/kg feed)	4.60 ± 0.57	6.48 ± 1.19^ab^	6.31 ± 0.99^ab^	6.02 ± 1.06
T5: As + Ab-Sp (1.5 gm/kg feed)	4.65 ± 0.61	6.09 ± 0.81^abc^	6.83 ± 1.61^ab^	6.13 ± 1.10
T6: As + Ab-Sp (2 gm/kg feed)	4.59 ± 0.33	4.90 ± 1.45^c^	6.19 ± 0.41^ab^	6.38 ± 1.84
T7: As + Com-Sp (1 gm/kg feed)	4.63 ± 0.35	5.46 ± 0.45^bc^	5.27 ± 0.49^b^	5.73 ± 1.01
T8: As + Com-Sp (1.5 gm/kg feed)	4.71 ± 0.38	6.03 ± 0.64^abc^	5.67 ± 0.59^ab^	7.14 ± 2.23
T9: As + Com-Sp (2 gm/kg feed)	4.61 ± 0.17	7.13 ± 0.68^a^	7.37 ± 0.38^a^	6.77 ± 1.31
Level of significance	ND	**	**	NS

Mean ± standard deviation are shown; ND signifies no analysis and NS denotes no significance. Within a column, values with comparable superscripts or no superscripts do not differ considerably, whereas values with differing superscripts differ considerably.

** Significance at the 1% level of probability.

### Serum MDA

**Figure 3.**
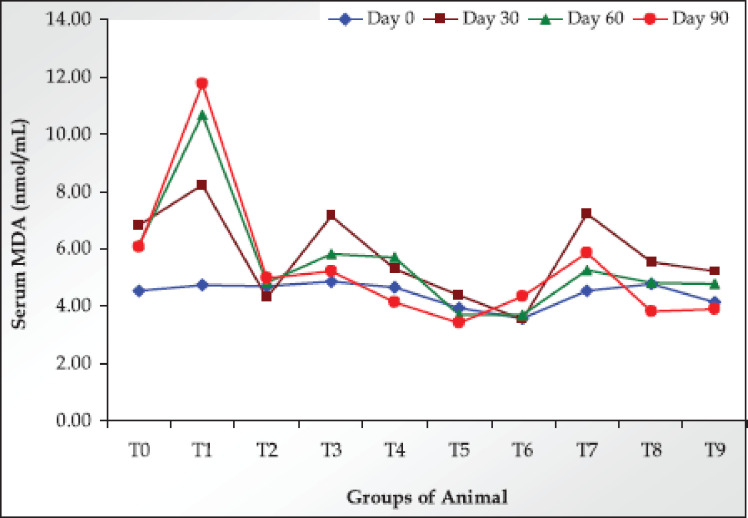
Values of serum MDA in the trial rats on different sampling days.

On day 0, the serum MDA values range from 3.57 ± 0.51 to 4.84 ± 0.78 among all the groups. As treatment significantly increased MDA values. However, both the Ab-Sp and Com-Sp treatments significantly decreased MDA values in a dose-dependent manner throughout the study (Fig. 3; Table 2).

### Serum MDA per mg of TSP

**Figure 4.**
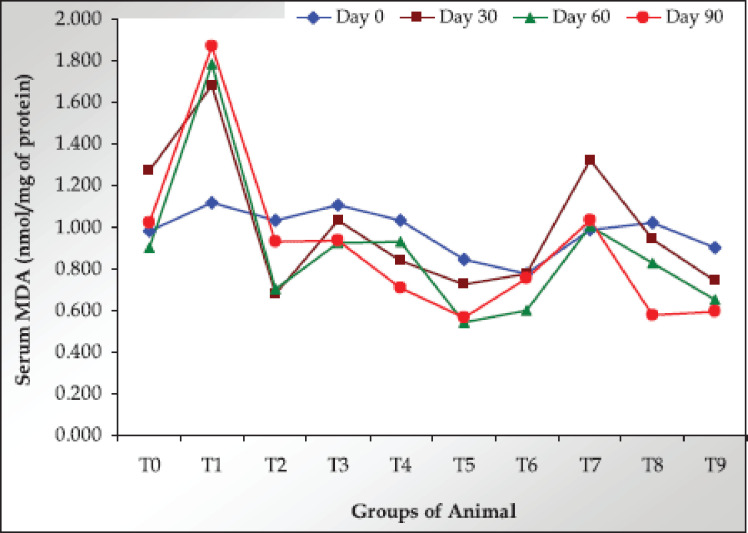
Values of serum MDA/mg of TSP in the trial rats on different sampling days.

The serum MDA/mg of TSP values were found highest in the As-treated group from day 30 to day 90. However, both the Ab-Sp and Com-Sp treatments significantly decreased MDA/mg of TSP values in a dose-dependent manner throughout the study. Like serum MDA, the Ab-Sp reduced MDA/mg of TSP better than the Com-Sp (Fig. 4; Table 3).

**Table 2.** Values of serum MDA in the trial rats on different sampling days.

Groups of animal	Serum MDA (nmol/ml)			
	Day 0	Day 30	Day 60	Day 90
T0: Control	4.52 ± 0.32	6.82 ± 1.67^abc^	6.12 ± 0.87^b^	6.07 ± 0.34^b^
T1: As 3 mg/kg BW (% of control)	4.75 ± 0.89	8.21 ± 0.63^a^ (20.38)	10.65 ± 1.43^a^ (74.02)	11.74 ± 0.92^a^ (93.41)
T2: Ab-Sp (2 gm/kg feed) (% of control)	4.69 ± 0.87	4.28 ± 0.13^de^ (–37.24)	4.81 ± 0.20^bc^ (–21.41)	4.98 ± 0.12^bc^ (–17.96)
T3: Com-Sp (2 gm/kg feed) (% of control)	4.84 ± 0.78	7.16 ± 0.63^ab^ (4.99)	5.83 ± 0.16^b^ (–4.74)	5.23 ± 1.68^bc^ (–13.84)
T4: As + Ab-Sp (1 gm/kg feed) (% of As control)	4.65 ± 0.68	5.30 ± 0.65^bcd^ (35.44)	5.70 ± 0.74^b^ (46.48)	4.15 ± 0.63^cd^ (64.65)
T5: As + Ab-Sp (1.5 gm/kg feed) (% of As control)	3.94 ± 0.76	4.37 ± 0.65^de^ (46.77)	3.71 ± 1.22^c^ (65.16)	3.43 ± 0.48^d^ (70.78)
T6: As + Ab-Sp (2 gm/kg feed) (% of As control)	3.57 ± 0.51	3.52 ± 0.46^e^ (57.13)	3.71 ± 0.94^c^ (65.16)	4.35 ± 1.00^cd^ (62.95)
T7: As + Com-Sp (1 gm/kg feed) (% of As control)	4.55 ± 0.24	7.22 ± 0.78^ab^ (12.06)	5.24 ± 0.33^bc^ (50.80)	5.85 ± 0.74^b^ (50.17)
T8: As + Com-Sp (1.5 gm/kg feed) (% of As control)	4.77 ± 0.09	5.54 ± 1.85^bcd^ (32.52)	4.80 ± 1.88^bc^ (54.93)	3.80 ± 0.41^cd^ (67.63)
T9: As + Com-Sp (2 gm/kg feed) (% of As control)	4.14 ± 0.56	5.21 ± 1.02^bcd^ (36.54)	4.77 ± 1.08^bc^ (55.21)	3.91 ± 0.45^cd^ (66.70)
Level of significance	ND	**	**	**

Values represent the mean ± standard deviation; values enclosed in parenthesis represent the percentage value; values enclosed in parenthesis without any sign represent the increased percentage value; and values enclosed in parenthesis without any sign represent the increased percentage value. Values with a “–” sign in parenthesis suggest a lower percentage value. Values with identical or no superscripts do not differ considerably within a column, but values with differing superscripts do.

** Significant at the 1% level of probability.

## Discussion

By measuring serum MDA and TSP, we were able to determine the degree of lipid peroxidation and the oxidative stress caused by chronic As exposure and the protective effects of spirulina (Ab-Sp and Com-Sp). This study discovered that As feeding increased serum MDA levels. Both types of spirulina (Ab-Sp and Com-Sp) significantly reduced them. The Ab-Sp (2.0 gm/kg feed) was the most effective in preventing lipid peroxidation caused by inorganic As toxicity.

The serum MDA levels were increased in the present study by As feeding and reached the highest levels in the experimental groups, increasing the trend from the start to the end of the trial. This result indicates that As feeding resulted in an increase in lipid peroxidation in the treated rat, implying an increased production of free radicals. Our findings corroborate a previous report that high As exposure was associated with increased lipid peroxide levels in the blood [9]. Both Ab-Sp and Com-Sp treatment significantly decreased serum MDA levels compared to the control and As-treatment groups. This result indicates that lipid peroxidation was improved with Ab-Sp and Com-Sp treatments alone and returned to the control level after the trial.

Similarly, MDA levels were found to be lower in all of the As and both spirulina-treated groups on all sampling days. However, the Ab-Sp significantly reduced serum MDA levels more than the Com-Sp (Table 2). The serum MDA/mg of TSP showed nearly identical scenarios to those observed in the serum MDA (Table 3). Spirulina’s antioxidant properties have been demonstrated through its extract’s inhibition of lipid peroxidation [21]. Spirulina is high in beta-carotene and vitamin E [22], and beta-carotene is one of the most effective antioxidants against free radicals that damage cells and cause cancer. As a result, supplementation with beta-carotene and vitamin E has been shown to reduce lipid peroxidation significantly [23].

The data indicate that serum TSP values varied significantly in the majority of samples. NaAsO_2_ treatment decreases TSP on day 30 and day 60, consistent with Rahman et al. [24], who found that the total protein content of arsenicosis patients’ serum was significantly lower than that of age-matched control groups in Bangladesh. Both spirulina increased TSP in a dose-dependent manner, with Ab-Sp demonstrating greater efficacy than commercial spirulina. However, increased TSP concentrations have been reported [25]. The results indicated that arsenicosis affected the MDA levels in the serum and the serum proteins of the patients.

**Table 3.** Values of serum MDA/mg of TSP in the trial rats on different sampling days.

Groups of animal	MDA (nmol/ml)/mg of TSP			
	Day 0	Day 30	Day 60	Day 90
T0: Control	0.981 ± 0.109	1.269 ± 0.443^ab^	0.901 ± 0.205^bcd^	1.019 ± 0.055^b^
T1: As 3 mg/kg BW (% of control)	1.118 ± 0.223	1.680 ± 0.130^a^ (32.39)	1.786 ± 0.344^a^ (98.22)	1.869 ± 0.185^a^ (83.42)
T2: Ab-Sp (2 gm/kg feed) (% of control)	1.030 ± 0.277	0.681 ± 0.085^c^ (–46.34)	0.700 ± 0.088^bcd^ (–22.31)	0.929 ± 0.313^bc^ (–8.83)
T3: Com-Sp (2 gm/kg feed) (% of control)	1.108 ± 0.226	1.032 ± 0.078^bc^ (–18.68)	0.922 ± 0.038^bc^ (2.33)	0.936 ± 0.359^bc^ (–8.15)
T4: As + Ab-Sp (1 gm/kg feed) (% of As control)	1.031 ± 0.265	0.836 ± 0.171^c^ (50.24)	0.929 ± 0.274^bc^ (47.98)	0.708 ± 0.187^bc^ (62.12)
T5: As + Ab-Sp (1.5 gm/kg feed) (% of As control)	0.843 ± 0.062	0.725 ± 0.140^c^ (56.85)	0.539 ± 0.095^d^ (69.82)	0.562 ± 0.031^c^ (69.93)
T6: As + Ab-Sp (2 gm/kg feed) (% of As control)	0.775 ± 0.064	0.773 ± 0.280^c^ (53.99)	0.599 ± 0.151^cd^ (66.46)	0.753 ± 0.374^bc^ (59.71)
T7: As + Com-Sp (1 gm/kg feed) (% of As control)	0.984 ± 0.057	1.320 ± 0.038^ab^ (21.43)	1.001 ± 0.130^b^ (43.95)	1.029 ± 0.133^b^ (44.94)
T8: As + Com-Sp (1.5 gm/kg feed) (% of As control)	1.019 ± 0.086	0.941 ± 0.384^bc^ (43.99)	0.829 ± 0.237^bcd^ (53.58)	0.574 ± 0.201^c^ (69.29)
T9: As + Com-Sp (2 gm/kg feed) (% of As control)	0.902 ± 0.153	0.743 ± 0.206^c^ (55.77)	0.651 ± 0.166^bcd^ (63.55)	0.595 ± 0.146^bc^ (68.16)
Level of significance	ND	**	**	**

The values show the mean standard deviation, whereas the figures in brackets show the percentage value. Figures in parenthesis without a sign indicate a higher percentage value; figures in parentheses with a “–” sign indicate a lower percentage value; ND = Not done analysis. Within a column, figures with comparable superscripts or no superscripts do not differ considerably, whereas figures with differing superscripts do (as per DMRT).

** Significant at the 1% level of probability.

## Conclusion

Our findings indicated that As supplementation increased serum MDA levels and decreased TSP levels, indicating increased lipid peroxidation and oxidative stress. However, both types of spirulina significantly improved this condition, most likely through a reduction in oxidative stress. The highest dose of Ab-Sp (2.0 gm/kg feed) was found to be the most effective in preventing lipid peroxidation in rats treated with inorganic As. As a result, Ab-Sp may be a natural, cost-effective, and safe method of reducing As toxicity.

## List of abbreviations

As: Arsenic; Ab-Sp: Agro-based spirulina; Com-Sp: Comercial spirulina; MDA: malondialdehyde; TSP: total serum protein; ROS: reactive oxygen species; NaAsO_2_: sodium arsenite; DW: deionized water; h: hour; min: minute; kg: kilogram; gm: grams.
